# When males outlive females: Sex‐specific effects of temperature on lifespan in a cyclic parthenogen

**DOI:** 10.1002/ece3.4473

**Published:** 2018-09-12

**Authors:** Barbara Pietrzak, Małgorzata Grzesiuk, Julia Dorosz, Andrzej Mikulski

**Affiliations:** ^1^ Department of Hydrobiology Faculty of Biology University of Warsaw at Biological and Chemical Research Centre Warsaw Poland

**Keywords:** cyclic parthenogen, *Daphnia*, longevity, phenotypic plasticity, sex, temperature

## Abstract

Lifespans of males and females frequently differ as a consequence of different life history strategies adopted to maximize fitness. It is well visible in cyclic parthenogens, such as water fleas of the genus *Daphnia*, where males appear in the population usually only for periods when receptive females are available. Moreover, even within one sex, different life history strategies and mechanisms regulating lifespan may exist. Previous studies suggested that *Daphnia* males may regulate their lifespan by staying in colder waters than females. We hypothesize that such behavioral mechanism should be associated with stronger reaction to low temperature–that is greater lifespan extension in males than in females. In this study, we monitored survivorship of *Daphnia magna* females and males of three clonal lines cultured at 16 or 20°C. The results did not provide a species‐level corroboration of our hypothesis; instead, they revealed very strong intraspecific differences in the responses of male and female lifespan to temperature change. They further suggest the existence of parallel life history strategies, hypothesis whose tests would bring new insights into the ecology of males in cyclic parthenogens.

## 1 INTRODUCTION

One of the most obvious consequences of the evolution of sexual reproduction is phenotypic differentiation between males and females. Beside basic differences in gonad structure and function, or in overall morphology, males and females differ in their strategies for reaching maximum fitness. These involve conspicuous differences in their physiology, behavior and life history, including rates of aging and physiological lifespan, that is, longevity under conditions of no extrinsic mortality. In other words, as males and females resolve life history tradeoffs differently, their optima for lifespan differ (Maklakov & Lummaa, 2013). Thus, as a result of different strategies adopted, the relative longevity of the sexes may be reversed even in closely related taxa (e.g., Hazzard, 1990; Promislow, Fedorka, & Burger, 2006). Moreover, even within a species, sexual dimorphism in lifespan has been experimentally shown to evolve predictably, according to environmental hazards, and rapidly, in a score of generations (Chen & Maklakov, 2014).

As the force of selection weakens with age in most animal species (Hamilton, 1966; but see Jones et al., 2014), physiological lifespan is more often an indirect result of selection pressures acting on other life history traits, on early fitness components, and less often a result of a more direct selection on long lifespan. Longevity differences often appear as a consequence of different costs of reproduction, which may differ substantially between the sexes. These costs include behavioral and morphological attributes associated with reproduction which may, for example, attract predators, limit ability to escape, or induce physiological cost impairing immunity, and thus, increase the mortality rates of individuals of one sex only (e.g., Fedorka, Zuk, & Mousseau, 2004; Han, Jablonski, & Brooks, 2015; Sommer, 2000). Nevertheless, there are cases where reproduction at older ages contributes to overall fitness of one sex enough to promote its longevity. This may be related to body size where, for example, larger older females produce larger broods (Congdon et al., 2003), or to female mate choice where, for example, females prefer older males (Beck & Powell, 2000).

Another interesting case is when only one of the sexes experiences periodicity of reproduction, like in cyclic parthenogens, where males appear only periodically, once in a few or many generations. In such a case, surviving to the next period when sexual females are available may be both challenging and rewarding for a male. Hence, there are two plausible scenarios of male appearance in such a population, and their potential lifespan can be expected to vary accordingly. First, males born when or shortly before the time sexual females appear can invest in early maturation and in fertilizing these available receptive females. Second, males born between the periods of the appearance of sexual females, or males outliving such a period, wait for the next opportunity to reproduce. Extending lifespan would, therefore, be highly advantageous. A long life would be a particularly profitable strategy in habitats where periods when receptive females appear are frequent.

The freshwater crustacean *Daphnia* is a cyclic parthenogen and a model organism widely used to investigate phenotypic plasticity (Ebert, Yampolsky, & Van Noordwijk, 1993; Lampert, 2011). A single genotype can express a multitude of phenotypes depending on the environment it experiences (see e.g., Colbourne et al., 2011; Tollrian, 1995), and also sex is environmentally determined. Although males and females are genetically identical (Hebert & Ward, 1972; LeBlanc & Medlock, 2015), they differ in morphology and physiology (MacArthur & Baillie, 1929a,b; Mitchell, 2001). Also, the sexes seem to show different life history strategies to maximize fitness. Females grow large, as the size of the brood chamber determines space available for eggs to develop (Bartosiewicz, Jabłoński, Kozłowski, & Maszczyk, 2015), and they seek resource rich and warm, although risky, surface waters (Lampert, 1989), where they grow fast. Males grow smaller than females (MacArthur & Baillie, 1929a), as they do not rely on large body size or accumulated resources for reproductive success. They also keep to deep, cold, and safe strata, where available (see e.g., Brewer, 1998; Mikulski, Bernatowicz, Grzesiuk, Kloc, & Pijanowska, 2011).

Whether males may extend lifespan between periods of receptive females availability in natural habitats is not known. A number of studies showed that under laboratory conditions *Daphnia* males live substantially shorter than females (Duneau, Luijckx, Ruder, & Ebert, 2012; Korpelainen, 1986; MacArthur & Baillie, 1929a,b; Schwarzenberger, Christjani, & Wacker, 2014). Yet, numerous studies also showed *Daphnia* males are less sensitive than females to a variety of stressors, resulting in their lower lifespan reduction under stressful conditions (e.g., Euent, Menzel, & Steinberg, 2008; Ikuno, Matsumoto, Okubo, Itoi, & Sugita, 2008; Lürling & Beekman, 2006; Thompson, Gipson, & Hall, 2017). At last, Pietrzak, Bednarska, and Grzesiuk (2010) hypothesized that although under the same temperature the lifespan of males may be similar to or shorter than that of females, it can be extended by their choice of colder habitat in nature.

Temperature is a factor that has been excluded from most of the above‐mentioned laboratory tests, yet it is one of crucial importance for any ectotherm. Brewer (1998), Spaak and Boersma (2001) and Mikulski et al. (2011) observed *Daphnia* males choose deeper water strata than females and, in consequence, experience temperatures even 4°C colder than females. Similar differences lead to increased lifespan of planktonic ectotherms (e.g., rotifers: Bosque, Hernandez, Perez, Todolı, & Oltra, 2001; Johnston & Snell, 2016; copepods: Brugnano, Guglielmo, Ianora, & Zagami, 2009; Kiørboe, Ceballos, & Thygesen, 2015; cladocerans: Orcutt & Porter, 1984; Engert et al., 2013), both via physical effect of decreased metabolic rate and via changes in gene expression (Johnston & Snell, 2016; see also a review by Keil, Cummings, & de Magalhães, 2015). Indeed, *Daphnia* choosing deeper, and thus colder, strata have been shown to live longer (Pietrzak et al., 2013).

As proposed above, there are two plausible life history strategies of males in a cyclic parthenogen: the short and the long lived. The switch may be triggered during development, thus, early determining the life course trajectory, or may be regulated behaviorally throughout life. If *Daphnia* males staying in colder water is indeed part of a functioning behavioral mechanism of actively extending their lifespan, physiological mechanisms triggered in the male phenotype and facilitating such response have likely evolved. We thus hypothesize that the effect of temperature on lifespan is stronger in males than in females. In consequence, males that live similarly long to females in warm surface water, may live longer than females in colder strata. There is some evidence supporting our hypothesis. MacArthur and Baillie (1929a,b) have already found that males’ metabolic rate and lifespan are affected by temperature more strongly than that of females.

## 2 MATERIALS AND METHODS

### 2.1 Study animals

**Figure 1:**
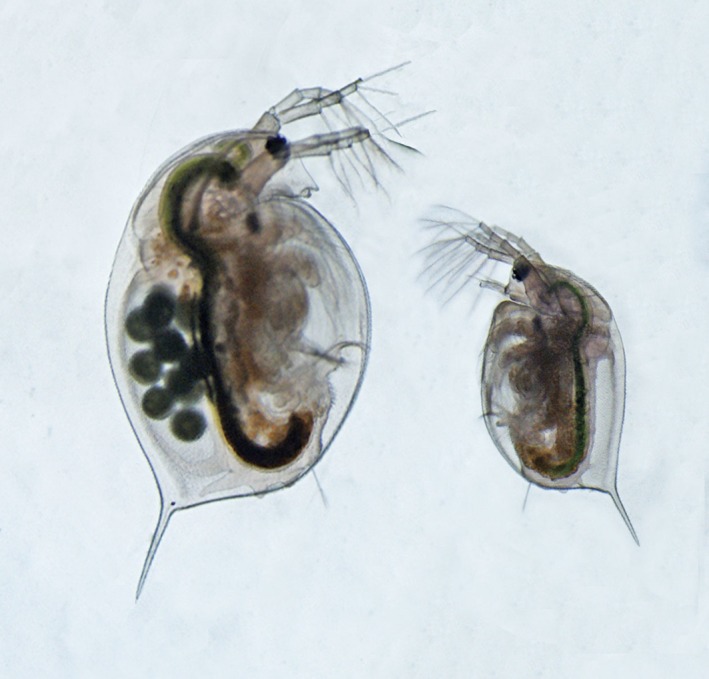
A female and a male of *Daphnia magna*

Twelve clonal lineages of *Daphnia magna* (Figure 1) were obtained from dormant eggs originating from three small water bodies in Poland, two from Belgium, and from Grosser Binnensee in Germany. *Daphnia* were cultured under constant conditions: density of 20 inds./L in filtered lake water changed every second day under constant dim light and at 18°C. Animals were fed with *Chlamydomonas reinhardtii* in concentration equivalent to 1 mg C_org_/L applied daily. They were cultured so in three replicates for three generations to eliminate interclonal differentiation caused by maternal effect (Mikulski & Pijanowska, 2009; Walsh, Whittington, & Funkhouser, 2014). Neonates from the second clutch established every next generation.

Male production was induced in the fourth generation using natural cues (as opposed to artificial chemical stimulation via application of, for example, methyl farnesoate (Lampert, Lampert, & Larsson, 2012)). As conditions that trigger production of males might also induce expression of other traits, all clones had to undergo the same procedure, despite the fact that in clones originating from different habitats sexual reproduction is likely triggered by a different set of environmental cues (see Macháček, Vaníčková, Seďa, Cordellier, & Schwenk, 2013, and references therein). A uniform procedure based on a season‐independent cue – crowding – was determined in the preliminary experiments conducted using the clones collected for the study. Females from the fourth generation were crowded, that is, cultured in a density of 35 individuals per 0.8 L, in replicate containers, other conditions remaining unchanged. Neonates from the first clutch of these *Daphnia* were separated from their mothers and their sex was determined.

Females belonging to only three clones released male neonates in sufficient numbers to proceed with the experiment. The first clone originated form Książęca pond (henceforth referred to as clone K), which is a small astatic reservoir with a concrete bottom located in Warsaw (Poland). The second clone came from Tersaert pond (henceforth referred to as clone T), a small water body located in Belgium. The third clone originated from Grosser Binnensee (henceforth referred to as clone B), which is a large shallow coastal lake situated in Germany.

### 2.2 Experimental design and procedures

Experimental individuals of both sexes were collected from the same mother cohort. All males from each mother were distributed individually into 100 ml glass vials not later than 6 hr after birth, and they were randomly assigned to one of the two temperature treatments. Simultaneously, for each clone, newborn females in a number equal to the number of males in that clone were distributed individually into 100 ml glass vials and randomly assigned to the treatments following the same procedure as was used for males. Altogether 122 neonate individuals were set, 28–33 per each sex‐temperature treatment. The warmer bath was kept at 20°C, the cooler was at 16°C, and these temperatures were set according to temperatures chosen by males and females in a depth selection experiment (Mikulski et al., 2011). Temperature was maintained using thermostats (Haake) and additional water stirrers were installed to improve the circulation of water and to ensure temperature homogeneity over the entire tanks.

The animals were kept in the vials in 100 ml filtered lake water and fed 1 mg C_org_/L *C*. *reinhardtii* daily. The food concentration applied in the experiment was above the incipient limiting level (Bohrer & Lampert, 1988). All animals got the same amount of food regardless of size or sex, consistent with other similar studies (e.g., Pietrzak, Bednarska, et al., 2010). The water was changed once before the first reproduction of females, approximately on the third day of life of each individual, and subsequently after every neonate release and moulting. Each offspring clutch was removed from the vial. The change of water in vials containing males was synchronized with the change of water in the vial containing their paired female (for that reason, at the start of the experiment, each male was randomly assigned to a single female from the same clonal cohort and the same temperature treatment). Lifespan of each individual was monitored every 24 hr. The experiment lasted for 110 days – until all animals in the warmer water (20°C) and a majority of those in the colder water (16°C) died.

### 2.3 Statistical analyses

Cox proportional hazards model was fit to the data (*coxph* in *survival* R package) to establish the relevance of sex and temperature and their interaction as variables explaining differences in lifespan. The interaction of sex and temperature was expected as a signature of males and females reacting differently to temperature. Wald test was used to check the significance of the estimated coefficients, and to test the omnibus null hypothesis that all of the coefficients are 0. Cox regression is considered insensitive to sample size and the type of the data distribution (see Bradley, 1974), and it takes into account the expected longevity of the animals surviving until the end of the experiment (right censored data).

The fit of the simple model (sex × temperature) was compared with the fit of the full model including clonal origin as one of the main effects and all interactions (clone × sex × temperature). To choose the best fit model, Akaike information criterion was used as the relative measure of fit and the relative likelihood of each candidate model *i* in the set was calculated as *exp*((AIC_min_−AIC_*i*_)/2) (Supporting information Table S1).

Survival curves were constructed using Kaplan–Meier estimator and compared with a log‐rank test (*survdiff* function at *ρ* = 0, *OIsurv* R package). Sequential Bonferroni method was applied to correct for multiple testing and *p*‐values were adjusted according to Holm (1979).

As juvenile mortality was observed in some cohorts, we performed an analogous analysis for adults only, to separate the early age effects from aging processes. Yet, as these results lead to similar conclusions as the above analysis, and are subject to an error resulting from the choice of the cut‐off age, as cohorts differed in the age at maturity, we do not present these results here.

## 3 RESULTS

**Figure 2:**
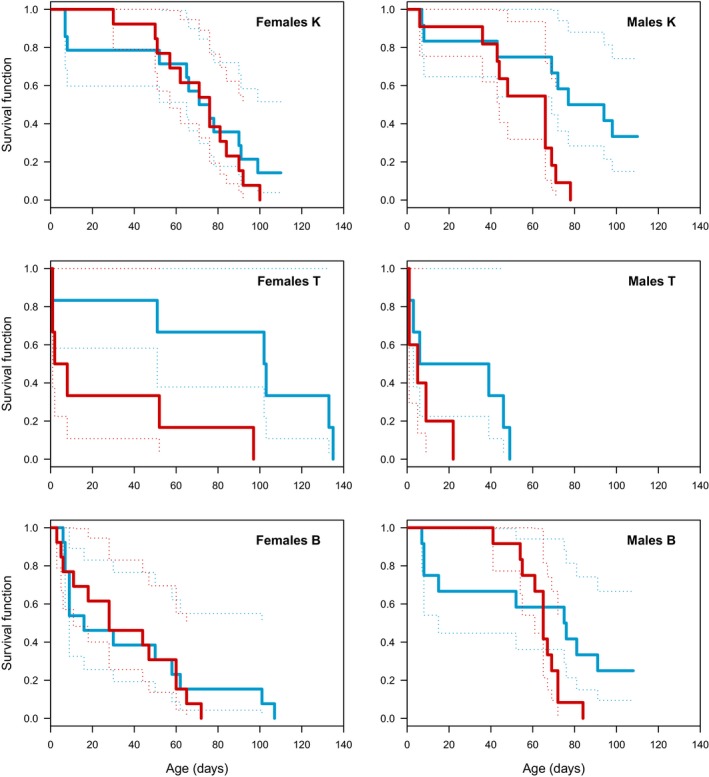
Kaplan–Meier estimates of survival and their 95% CI (blue: 16°, red: 20°C) for females (left) and males (right) of K, T and B *Daphnia magna* clones (top, middle, and bottom, respectively)

Fitting Cox proportional hazards model to the data revealed strong effects of clonal origin on survival (Supporting information Table S1), hence, the data were hereafter analyzed for each clone separately. The differences between males and females in their reaction to temperature varied between clones, still, the maximum observed lifespan was longer at 16 than at 20°C in all tested clones (Figure 2).

Only in clone K, the effect of temperature on lifespan was significantly larger in males than in females (temperature × sex interaction, Table 1). Survival curves for treatments at 16 and 20°C differed in males (χ^2^ = 7.2, *df* = 1, *p* = 0.044) (all presented *p*‐values are the corrected values, see Methods), with male median lifespan of 86 and 66 days, respectively, but they did not differ in females (χ^2^ = 0.5, *df* = 1, *p* = 1), with median lifespan of 76 and 74 days, at 16 and 20°C, respectively (Figure 2).

**Table 1:** Cox proportional hazards estimates of the determinants of age at death in three clones of *Daphnia magna*. Asterisk marks p<0.05

Variable	Clone K			Clone T			Clone B		
	Coeff	*z*	*p*	Coeff	*z*	*p*	Coeff	*z*	*p*
Sex	−0.55	−1.20	0.230	2.15	2.41	0.016*	−1.05	−2.38	0.0173*
Temp	0.26	0.63	0.527	1.85	2.24	0.025*	0.39	0.94	0.3453
Sex × Temp	1.36	2.14	0.033*	−1.33	−1.29	0.198	0.23	0.39	0.6976
	W	*df*		W	*df*		W	*df*	
Wald test	10.7	3	0.013*	8.25	3	0.041*	10.8	3	0.0128*

Higher temperature decreased survival of individuals from clone T, and males lived shorter irrespective of temperature (Table 1), yet, neither in males nor in females did the survival curves differ significantly between temperature treatments (males: χ^2^ = 2, *df* = 1, *p* = 0.477, females: χ^2^ = 4.9, *df* = 1, *p* = 0.136). Median lifespan of males was 23 days at 16°C and 5 days at 20°C, in females it was 102 and 5 days, respectively.

Only sex affected survival in clone B. Males in this clone had higher survival rates than females, irrespective of temperature (Table 1), with male median lifespan of 76 days at 16°C and 65 days at 20°C, and female median lifespan of 28 and 16 days, respectively. Neither in males nor in females did the survival curves differ significantly between temperature treatments (males: χ^2^ = 2.6, *df* = 1, *p* = 0.416, females: χ^2^ = 0.2, *df* = 1, *p* = 1).

## 4 DISCUSSION

The results presented in this study showed large differences between genotypes in the response of the sexes to temperature, and hence, we analyzed the data for each clone separately. We discuss both the observed strong genotype effects and the particular clonal responses below.

The temperature effect on lifespan is not merely a passive process, but is under gene regulation (Johnston & Snell, 2016; Keil et al., 2015; Xiao et al., 2013). Hence, intraspecific differences in how genotypes and phenotypes, here sexes, respond in lifespan to temperature change are very much expected. Yet, they are still an understudied phenomenon. The inattention may stem from the fact that traditionally, the negative correlation between temperature and lifespan, or the positive correlation between temperature and aging rates have been linked to passive thermodynamic effects. Temperature governs metabolic rates, increasing them exponentially (Gillooly, Brown, West, Savage, & Charnov, 2001), and one who lives fast, lives short (e.g., Van Voorhies & Ward, 1999). In an early work of MacArthur and Baillie (1929a,b), the authors concluded that the patterns they observed in *Daphnia* reflected what would be expected if metabolic rates governed longevity. Lower temperature has been shown to act exponentially to prolong life in a parasitic wasp (McDougall & Mills, 1997). After compiling data for as many as over 1,000 populations of a wide variety species, Munch and Salinas (2009) concluded, that much of the latitudinal variation in lifespan seen within species may be explained by temperature and within the “metabolic theory of ecology.” Under this rate of living paradigm, the accelerated aging observed under higher temperatures would simply reflect higher rates of age‐related damage accumulation (see commentary in Valenzano, Terzibasi, Cattaneo, Domenici, & Cellerino, 2006).

It has become clear, though, that the mechanisms behind the observed effects of temperature on lifespan surpass by far the mechanistic effects of temperature on the rate of biochemical reactions, and that active regulation of several genes and gene pathways is involved in the response to decreased temperature. Patterns of gene expression upon adaptation to cold have been studied in fish (Grace et al., 2004; Podrabsky & Somero, 2004). Genetic programs actively promoting longevity at cold temperatures have been revealed in *Caenorhabditis elegans* (Xiao et al., 2013), as well as a mutation in a single gene which flattens its lifespan response to temperature (Horikawa, Sural, Hsu, & Antebi, 2015). Zhang et al. (2016) showed temperature differentially regulates the nematode lifespan at different life stages via a single pathway. Results obtained in a recent study using rotifers suggested that multiple pathways are involved in temperature mediated life extension, among them a complex signaling cascade regulated by a sensory receptor (Johnston & Snell, 2016). In *Daphnia,* temperature changes patterns of heat shock protein expression in a sex‐specific manner (Mikulski et al., 2011), which may have consequences for their lifespans (Schumpert, Anderson, Dudycha, & Patel, 2016). Temperature thus exerts its effects not only via passive thermodynamics, but also via differential modulation of gene expression, and these effects are subject to selection and underlie adaptation.

While the intraspecific differences in the response of longevity to temperature manipulations have rarely been studied, other manipulations which possibly share with temperature mechanisms of action have been shown to induce strain‐specific effects. Different genetic isolates of a *Brachionus* rotifer (Gribble, Kaido, Jarvis, & Mark Welch, 2014) and *D*. *magna* (Pietrzak, Grzesiuk, & Bednarska, 2010) respond differently to intermittent fasting or caloric restriction, respectively. Their different responses to temperature manipulation might be expected, as there seems to be a link between nutrient and thermal sensing, metabolism, and longevity (Horikawa et al., 2015). Recently, Johnston and Snell (2016) observed that lifespans of closely related rotifer species respond differently to temperature manipulation. Even more closely, Henning‐Lucass, Cordellier, Streit, and Schwenk (2016), studying the response to temperature in *Daphnia galeata* clones of different age (i.e., hatched from resting eggs of different age), found no overall effect of temperature on *Daphnia* survival rate, as individuals from clones of different age reacted differently.

In our study, clonal effects were very strong (Supporting information Table S1). Our hypothesis that male lifespan is more strongly affected by temperature than that of females was confirmed only for one of the three studied clones: only in clone K, the sex × temperature interaction term was significant, as predicted. The result suggests males of this genotype could plausibly extend their lifespan by seeking cooler temperatures, as expected. Greater sensitivity to temperature of small‐bodied males, in comparison with females, stays in agreement with the results of MacArthur and Baillie (1929a,b), although they did not observe a longevity switch between sexes. Meanwhile, in clone T temperature affected longevity of the individuals irrespective of their sex. Also, individuals of this clone were short‐lived in general, in comparison with other clones, with increased death rates at younger ages in particular, possibly an uncontrolled factor introducing additional mortality being at play. Nevertheless, T clone males seemingly lived shorter than females, regardless of temperature, which would be similar to observations on the same species made by Korpelainen (1986) and Schwarzenberger et al. (2014). At last, in clone B, males had higher survival rates than females, irrespective of temperature. This is in turn consistent with expectations of Pietrzak, Bednarska, et al. (2010), who already suggested that *D. magna* males from Binnensee might live longer than females in their natural habitat. Previous inconsistencies concerning *Daphnia* male longevity could thus be due to interclonal differences evidenced here.

At last, our results suggest the mechanism behind lifespan divergence of the sexes may be twofold: temperature independent or dependent. Where it is independent, individuals of one sex live longer irrespective of temperature, such as males of clone B in this study. Where it is temperature dependent, individual survival of one sex increases more strongly upon temperature decrease, as it does in K clone males staying in colder water. Indeed, in their natural habitats males of several *Daphnia* species have been found to stay in colder waters than females by staying deeper in the water column (Brewer, 1998; Macháček et al., 2013; Mikulski et al., 2011; Spaak & Boersma, 2001). Recently, Glaholt, Kennedy, Turner, Colbourne, and Shaw (2016) documented high variability in female thermal preferences within populations with a simultaneous lack of variation among studied populations. The authors suggested the existence of niche partitioning that could reduce intraspecific competition. Such a multitude of strategies or preferences is consistent with clone specificity in environmental cues that induce male production (seen and reviewed in Macháček et al., 2013).

Within and between habitat interclonal differences in type and strength of reaction in response to a variety of environmental conditions have often been observed in several *Daphnia* species (Bernatowicz & Pijanowska, 2011; De Meester, 1993, 1996; Reede & Ringelberg, 1995; Spitze, 1992; Weider, 1984), as well as interclonal differences in lifespan have (Dudycha & Hassel, 2013; Dudycha & Tessier, 1999). The question now arises if the reactions observed here in these particular *D. magna* clones can be adaptive in the specific environmental context of their habitat of origin. Another question will be if different clonal responses are shaped by selective pressures present in these habitats. Answering this question, however, would require clonal replicates within habitat. Here, three single genetic isolates (clones) from three water bodies were studied, which in the face of the lack of clonal replicates, cannot even be regarded as showing strategies representative for their habitats of origin.

Nevertheless, speculating on the adaptiveness of the particular patterns of responses observed here brings us to conclude they all may be adaptive and represent different strategies. On one hand, short lifespan, particularly in males, and their likely maturing early, would be adaptive in very short‐lived temporary habitats. Short male lifespan would also be adaptive in those temporal or permanent habitats that experience rare and very predictable (e.g., annual) collapse in environmental conditions, where upon the single event of sexual reproduction resting eggs are produced and active population recedes. Korpelainen (1986), who examined clones from small astatic rock pools, observed a similar strategy in *D. magna* males. On the other hand, wherever there are several periods when receptive females appear – even more so if they are highly unpredictable – extending male lifespan would be highly advantageous (see a parallel example of ticks, Davey, Osburn, & Castillo, 1983). Long‐lived males either born between the periods of the appearance of sexually reproducing females, or outliving such a period, could thus wait for the next opportunity to reproduce. In *Daphnia*, the production of males is usually triggered by less stringent cues than those that are needed to trigger sexual egg production (Hobæk & Larsson, 1990; Kleiven, Larsson, & Hobæk, 1992). The presence of mature long‐lived males in the population will be beneficial, as it allows to skip the phase of male production and thus shortens the time to the release of resting eggs.

In our experiments, we used clones originating from different water bodies; the variation in responses and strategies represented is thus not surprising. Moreover, the effect of this interclonal divergence was stronger than the other effects studied, that is, that of sex or temperature. The choice of these particular and only three clones resulted from our scrutiny and consistency in applying the same environmental cues to induce male production in all examined clones. Limited by the number of available males were also our experimental clonal cohorts. We used conservative tests and statistics insensitive to sample size to avoid any false positives. Still, by sampling three genetic isolates, we sampled three different survival strategies. It would need further experimental testing to see whether they cover the variety of survival strategies observed in *D. magna* males, or whether they are representative for their habitats of origin. And finally, although studies performed using single strains within species provide greater opportunity for straightforward answers and coherent conclusions (see e.g., Schwarzenberger et al., 2014; Schwartz, Pearson, Dawson, Allison, & Gohlke, 2016 for important recent examples), using several clonal lineages provides insight into the multitude of existing responses and strategies applied.

## 5 CONCLUSIONS

1. We did not provide a species‐level corroboration of our hypothesis of the differential effects of temperature on *Daphnia* male and female lifespan. Instead, we revealed very strong intraspecific differences in these responses, which again points to the necessity of experiments using multiple genotypes for proceeding with any ecological inference.
2. Our results suggest the possibility of parallel lifespan regulating mechanisms in *D*. *magna* males. Relative longevity of males may be temperature independent or dependent. Where it is temperature dependent, short‐lived at higher temperatures males are expected to live longer than females in thermally stratified habitats, as they choose deeper colder strata. Similar mechanisms could apply in other cyclic parthenogens.

## AUTHOR CONTRIBUTIONS

AM, BP, and MG generated the hypothesis and all authors designed the study; JM, MG, and AM collected the data; AM and BP conducted statistical analyses; BP and AM wrote the manuscript and all authors contributed to drafting, editing, and revising the manuscript. All authors read and approved the final manuscript.

## DATA ACCESSIBILITY

Dryad data accession number: https://doi.org/10.5061/dryad.tc736qr
