# Supplementary material for: When males outlive females: Sex‐specific effects of temperature on lifespan in a cyclic parthenogen
Source: Ecol Evol. 2018 Sep 12;8(19):9880–8. doi: 10.1002/ece3.4473 (PMC6202703; doi:10.1002/ece3.4473)
Supplement: Supplementary file 1 [file ECE3-8-9880-s001.docx]

Supplementary Table S1. Candidate models considered and the best-fit model (bold) based on Akaike Information Criterion (AIC) and the relative likelihood of the model (RL).

| Model | AIC | RL |
| --- | --- | --- |
| Sex*Temp | 905 | <0.00001 |
| Clone*Sex*Temp | **879** | **1.00000** |
